# Longitudinal residual strain and stress-strain relationship in rat small intestine

**DOI:** 10.1186/1475-925X-5-37

**Published:** 2006-06-07

**Authors:** Yanling Dou, Yanhua Fan, Jingbo Zhao, Hans Gregersen

**Affiliations:** 1 Clinical Institute, Aarhus University, Aarhus, Denmark; 2 Gastroenterology Department, China-Japan Friendship Hospital, Beijing, China; 3 Center for Visceral Biomechanics and Pain, Aalborg Hospital, Aalborg, Denmark; 4 Center for Sensory-Motor Interaction, Aalborg University, Aalborg, Denmark

## Abstract

**Background:**

To obtain a more detailed description of the stress-free state of the intestinal wall, longitudinal residual strain measurements are needed. Furthermore, data on longitudinal stress-strain relations in visceral organs are scarce. The present study aims to investigate the longitudinal residual strain and the longitudinal stress-strain relationship in the rat small intestine.

**Methods:**

The longitudinal zero-stress state was obtained by cutting tissue strips parallel to the longitudinal axis of the intestine. The longitudinal residual stress was characterized by a bending angle (unit: degrees per unit length and positive when bending outwards). Residual strain was computed from the change in dimensions between the zero-stress state and the no-load state. Longitudinal stresses and strains were computed from stretch experiments in the distal ileum at luminal pressures ranging from 0–4 cmH_2_O.

**Results:**

Large morphometric variations were found between the duodenum and ileum with the largest wall thickness and wall area in the duodenum and the largest inner circumference and luminal area in the distal ileum (p < 0.001). The bending angle did not differ between the duodenum and ileum (p > 0.5). The longitudinal residual strain was tensile at the serosal surface and compressive at the mucosal surface. Hence, the neutral axis was approximately in the mid-wall. The longitudinal residual strain and the bending angle was not uniform around the intestinal circumference and had the highest values on the mesenteric sides (p < 0.001). The stress-strain curves fitted well to the mono-exponential function with determination coefficients above 0.96. The *α *constant increased with the pressure, indicating the intestinal wall became stiffer in longitudinal direction when pressurized.

**Conclusion:**

Large longitudinal residual strains reside in the small intestine and showed circumferential variation. This indicates that the tissue is not uniform and cannot be treated as a homogenous material. The longitudinal stiffness of the intestinal wall increased with luminal pressure. Longitudinal residual strains must be taken into account in studies of gastrointestinal biomechanical properties.

## Background

The residual stress is the stress remaining in an organ when external forces are removed (no-load state) and residual strain is the deformation from the no-load state to the zero-stress state (the residual stress released), have important physiological implications. It makes the stress distribution more uniform throughout the organ wall, influences the contractile force of muscles in organs such as the heart and blood vessels, and influences the elasticity of the tissue because the stiffness of soft tissues increases with the stress [1]. Furthermore, the residual stress and strain may serve as growth-regulating factors [2] and may in the digestive tract protect the mucosa from injury during excessive loading by reducing the stress concentration in the mucosal layer [3]. Alterations in residual strain are caused by growth and remodeling of cells and extracellular matrix. Hence, study of the residual strains is a way to investigate structural remodeling [1]. Furthermore, mechanical analysis would benefit by using the zero-stress configuration as the reference state [2-4]. Research on residual strain in soft tissues has previously been done mainly in the cardiovascular system based on one- or two-dimensional measurements [5-7]. Three-dimensional (circumferential, longitudinal and radial) data on residual strain have been presented by Costa et al. [8] for the mid-anterior canine left ventricle.

In recent years, circumferential residual strains have been studied in various parts of the gastrointestinal tract. The first gastrointestinal data were obtained by Gregersen et al. [3] in guinea pig duodenum followed by studies in the rat esophagus [9], the rat colon [10], and the rat small intestine [11]. In the previous intestinal studies we observed that the intestine bends outwards in longitudinal direction when a radial cut is made [11]. Therefore, to obtain a more detailed description of the stress-free state of the intestinal wall, longitudinal residual strain measurements are needed. Furthermore, data on longitudinal stress-strain relations in visceral organs are scarce though some data exist for arteries and the esophagus [12-14]. For better understanding of the intestinal transport function, we also need to know the stresses and strains in multiple directions [15]. The present study aims to investigate the longitudinal residual strain and the longitudinal stress-strain relationship in the rat small intestine.

## Materials and methods

### Animals and tissue sampling

Ten female Wistar rats weighing approximately 230 g were included in this study. The experiments were done according to the Danish national guidelines for animal experimentation. The protocol was approved by the committee for animal experimentation.

The rats were anaesthetized with pentobarbital sodium (50 mg/kg ip) and a midline laparatomy was made. Papaverine (60 mg/kg) was injected into the lower thoracic aorta through an i.v. cannula (22 G/25 mm) to abolish contractile activity of smooth muscle. The whole duodenum and a 5-cm long segment of the distal ileum from 1 cm proximal to the ileo-caecal valve were dissected and excised. After gently clearing the residual contents in the lumen with saline, the segments were placed into cold calcium-free Krebs solution (put on the ice) containing 6% dextran and EGTA (0.25%) aerated with a gas mixture (95% O_2 _and 5% CO_2_, pH 7.4). Under a light microscope, two short segments, 3–4 mm in length from the descending duodenum distal to the bile duct were cut for the no-load and zero-stress state tests for acquiring longitudinal bending angle and residual strain data in the duodenum. Similarly, two short specimens of the ileum for the no-load and zero-stress state tests were cut and transferred to separate small organ baths containing Krebs solution. Another ileum segment, about 2–3 cm long, was used for the longitudinal stress-strain experiment. The stress-strain experiment was not performed in the duodenum.

### Biomechanical test

The following experiments were done at the room temperature in the Krebs solution.

#### The no-load and zero-stress state tests (Fig. 1a, 1b, 1c)

**Figure 1:**
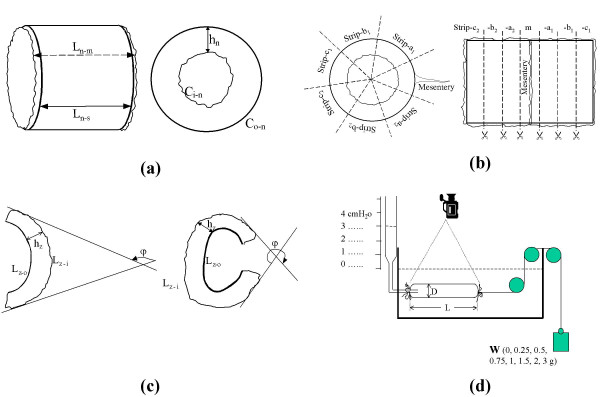
**A No-load state**. Left: Image of intestinal segments. Mucosal length (L_m_), serosal length (L_s_). n denotes the no-load state. Right: cross sectional image. Wall thickness (h), circumference (C), inner (mucosal) surface (i), and outer (serosal) surface (o). **B** Illustration of the way of cutting the tissue and labelling of the strips. Intestinal segments were cut into longitudinal strips and labelled according to their circumferential locations. Strip-m (strip at the mesenteric side), strip-a1 and strip-a2 (strips near the mesenteric side), strip-b1 and strip-b2 (strips near the anti-mesenteric side), strip-c1 and strip-c2 (strips at the anti-mesenteric side). **C** Longitudinal zero-stress state. Longitudinal strips tend to bend towards serosa. The zero-stress state is denoted by z, and the bending angle by φ **D** Longitudinal stress-strain experiment in rat small intestine. The illustration of the distension experimental set up on ileal segment. At each pressure of 0, 1, 2, 3 and 4 cmH2O, put weight of 0, 0.25, 05, 0.75, 1, 1.5, 2, and 3 gram on the distal end respectively. Three minutes were awaited to acquire equilibration after applying a combined intraluminal pressure and pulling force. D, L and W denote diameter, length and weight, respectively.

Cross-sectional and lateral images were taken of the intestinal specimens in the no-load state (Fig. 1a). Afterwards, the specimens were cut into seven 1–2-mm-wide longitudinal strips (Fig. 1b) to obtain the longitudinal zero-stress state. To reduce the possible affect of the width variation on the bending angle and residual strain, the 7 strips of similar width were cut from the segment. They were labelled according to their circumferential location: strip-m (strip at the mesenteric side); strip-a1, strip-a2 (strips near the mesentery); strip-b1, strip-b2 (strips near the opposite side of the mesentery); and strip-c1, strip-c2 (strips at the opposite side of the mesentery). Photographs were taken again about 1 hour after the cutting to allow viscoelastic creep to take place (Fig. 1c).

### The longitudinal stress-strain experiment (Fig. 1d)

The distal end of the segment from the distal ileum, 2–3 cm in length, was ligated and connected to a thread that passed a pulley block and was connected to hanging weights (0–3 grams). The proximal end of the intestinal segment was connected via a tube with a fluid-filled container level for applying pressures to the intestinal lumen. Before running the mechanical test, the tissue was preconditioned in order to obtain stable and repeatable stress-strain relations. The preconditioning was done by applying a 3 grams longitudinal force and 4 cmH_2_O intraluminal pressure to the intestinal segment for 2 min. The stress-strain test was started 5 minutes after the preconditioning. The pressure range was 0 to 4 cmH_2_O. At each pressure, the longitudinal tensile force was varied between 0 and 3 grams. The pressure range was selected because it corresponds to physiological pressures [15]. The physiological range for longitudinal forces in vivo is unknown. Therefore, we picked the same range of longitudinal weight as used in other intestinal motility experiments [16]. A combined intraluminal pressure and pulling force was applied and after three minutes photographs of the intestinal segment were taken.

### Histology

About 0.5 cm ileal segment close to the segment for distension was fixed in 10% buffered formalin over 24 h, Then, the specimen was dehydrated in a series of graded ethanol (70%, 96% and 99%) and embedded in paraffin. Five-micron sections were cut perpendicular to the mucosa surface and the paraffin was cleared from the slides with coconut oil (over 15 min 60°C). The sections were redehydrated in 99%, 96% and 70% ethanol followed by a 10-min wash in water and stained with hematoxylin and eosin. The thickness of the muscle layers and the height of the villus in all segments was measured by the same pathologist in a blinded review.

### Data analysis

The morphometric data were obtained from the digitized images of the photographs of segments in the zero-stress, no-load and stretched-pressurized states (Fig. 1). Using image analysis software (Optimas ver. 5.2, Optimas Corp., USA), the following data were measured from each specimen (illustrated in Fig. 1): the longitudinal length (L), the circumferential length (C), the wall thickness (h), the wall area (A), and the longitudinal bending angle at the zero-stress state (φ, degrees). Furthermore, the thickness and area of the villus and muscle layers were measured. The subscripts i, o, m, n, z and p refer to the inner (mucosal) surface, outer (serosal) surface, the mid-wall, the no-load state, zero-stress state and loaded condition. The bending angle (φ) was defined as the angle of the intestinal segment bend in longitudinal direction when a 1–2-mm-wide longitudinal strip was cut (Fig. 1b and 1c). Furthermore, the outer diameter (D) and the length (L) were measured from the images of the loaded segments.

The measured data was used for computation of biomechanical parameters [15] defined as:

The bending angle per unit length:



Longitudinal residual Green's strain at the mucosal surface:



Longitudinal residual Green's strain at the serosal surface:



The stress and strain in the distal ileum in the loaded state were determined under the assumptions that the intestinal wall was homogenous and the intestinal shape was circular and cylindrical. The calculations were based on knowing the no-load state dimensions, the outer diameter and length of the specimen at varying pressures, and assuming incompressibility of the intestinal wall. The longitudinal stretch ratio ; the circumferential stretch ratio  where ; the luminal radius, , where the *A*_*n *_is the cross-sectional area of the wall in the no-load state; the wall thickness, *h*_*p*_*= r*_*o*-*p*_*- r*_*i*-*p *_; the mucosal circumferential length, *C*_*i*-*p*_*= *2*πr*_*i*-*p *_; the serosal circumferential length,*C*_*o*-*p*_*= *2*πr*_*o*-*p*_; and the mid-wall circumferential length,  were computed.

The longitudinal Kirchhoff 's stress and Green's strain at a given loaded condition were computed according to the following equations [13]:

Longitudinal Kirchhoff's stress due to longitudinal force:



where *W *is the weight (g) (Fig. 1d).

Longitudinal Kirchhoff's stress due to distension:



Because of the nature of the villus structure, the villus layer unlikely carries tensile mechanical load. The wall thickness in the above equations did not include the thickness of the villus. The total longitudinal Kirchhoff's stress was defined as:

*S*_*l *_= *S*_1 _+ *S*_2 _    (6)

The mid-wall longitudinal Green's strain was defined as:



The no-load state was used as reference for the longitudinal strain because the mid-wall length did not change from no-load state to zero-stress state in the longitudinal direction.

It was tested how well the stress-strain curves fitted to the exponential function [13]:

*S *= (S* + *β*)*e*^*α*(E-E*) ^- *β *    (8)

using Tablecurve software (Jandel Scientific) where *α *and *β *are material constants and S* and E* are corresponding longitudinal kirchhoff stress and Green strain values obtained arbitrarily.

### Statistical analysis

The data were representative of a normal distribution and accordingly the results were expressed as mean ± SE. The constants *α *and *β *obtained from the non-linear fitting of stress-strain curves were used for the statistical evaluation of the stress-strain data. Due to the symmetry between a1 and a2, b1 and b2, and c1 and c2, respectively, they were combined into location a (next to the mesentery), b (intermediate) and c (the opposite side of the mesentery). Two-way ANOVA was employed to detect the differences between locations a, b, c and m and between the axial locations (duodenum and ileum) (Sigmastat 2.0™). In case of significance, data were evaluated in pairs by a multiple comparison procedure (Student-Newman-Keuls method). Spearman's correlation test was used to demonstrate possible association between the biomechanical and morphometric parameters. The results were regarded as significant when p < 0.05.

## Results

**Figure 2:**
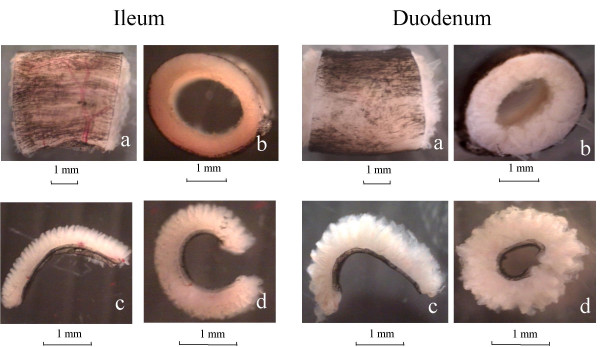
**Photograph of specimen from the distal ileum(left) and duodenum (right) in small organ bath containing the Krebs solution**. Top: the no-load state of the intestinal segment (about 3–4 mm in length) with longitudinal image (a) and cross-sectional image (b). Bottom: the longitudinal zero-stress state was obtained by cutting the segment (a) into longitudinal strips. The bending angle φ < 180° (c) and >180° (d).

Figure 2 depicts a specimen from the distal ileum and duodenum cut into 3–4 mm-long segment (a) to obtain the no-load state (b). After cutting the specimens into longitudinal strips, the strips bent outwards into a configuration with a bending angle in the longitudinal direction (c, d).

The morphometric data from the duodenum and distal ileum in the no-load state are shown in Table 1. Compared to the duodenum, the ileum had a bigger inner circumferential length and luminal area, and smaller wall thickness and wall area (Figure 2, p < 0.001).

**Table 1:** Morphometric data obtained from the descending duodenum and distal ileum in the no-load state

**Parameters**	**Duodenum**	**Ileum**	*p *Value*
Inner circumferential length	5.1 ± 0.3 (mm)	7.6 ± 0.2 (mm)	< 0.001
Outer circumferential length	9.8 ± 0.3 (mm)	10.8 ± 0.3 (mm)	> 0.05
Wall thickness	0.76 ± 0.02 (mm)	0.51 ± 0.02 (mm)	< 0.001
Luminal area	1.9 ± 0.2 (mm^2^)	4.60 ± 0.2 (mm^2^)	< 0.001
Wall area	6.2 ± 0.3 (mm^2^)	4.51 ± 0.2 (mm^2^)	< 0.001

Values are means ± SE. N = 10. * student *t*-test between duodenum and ileum.

**Figure 3:**
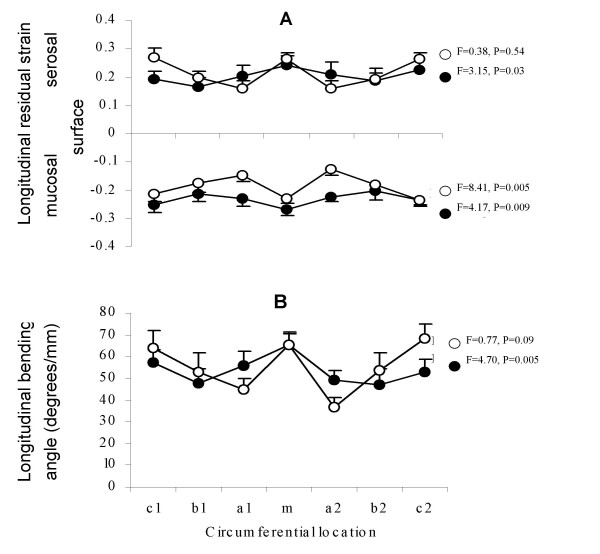
**longitudinal residual strain and longitudinal bending angle**. Mucosal and serosal longitudinal residual strain (A) and longitudinal bending angle (B) as function of circumferential locations (m, a, b, and c) in the duodenum (●) and ileum (◯).m, mesenteric side; a1 and a2, locations near the mesenteric side; b1 and b2, locations near the anti-mesenteric side; c1 and c2, the anti-mesenteric side. Values are mean ± SE. F and P represent statistical results from two-way ANOVA of data on axial direction (duodenum and ileum) and circumferential direction (a, b, c and m), respectively.

The distribution of the longitudinal residual strain and longitudinal bending angle around the intestinal circumference in the duodenum and ileum are shown in figure 3. The average bending angle did not differ between the duodenum and ileum (55.2 ± 2.9 vs. 56.4 ± 2.9°/mm, p > 0.05). The longitudinal residual strain was tensile at the serosal surface with values of 0.21 ± 0.01 in duodenum and 0.22 ± 0.01in ileum, and compressive at the mucosal surface with values of -0.24 ± 0.01 in the duodenum and -0.19 ± 0.01in the ileum. The longitudinal residual strain at the mucosal side was different between the duodenum and ileum (p < 0.01) but did not differ at the serosal side (p > 0.5). The absolute value of longitudinal residual strain (both at the mucosal and serosal surface) and the longitudinal bending angle were larger on the mesenteric (both for duodenum and ileum) and anti-mesenteric sides (only for ileum) when compared to strips from the remaining circumference (p < 0.001). Statistical correlation was found between the longitudinal bending angle and longitudinal residual strains at both serosal (r = 0.84, p < 0.001) and mucosal surfaces (r = 0.52, p < 0.001). The bigger is the longitudinal bending angle, the bigger is the serosal longitudinal residual strain and the more negative mucosal longitudinal residual strain.

**Figure 4:**
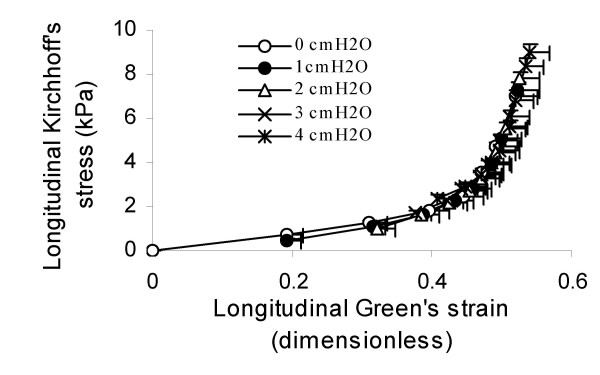
**Longitudinal stress-strain relationships at different luminal pressures**. Values are mean ± SE. The constants from the curve fitting appear in Table 2.

The longitudinal stress-strain relationships in the distal ileum are shown in figure 4. The data fitted well to the mono-exponential function with determination coefficients above 0.96. *α *and *β *values including statistics are provided in Table 2. The *α *constant increased and *β *constant decreased as function of pressure, indicating increased stiffness with pressure. When a load of 4 cmH_2_O and longitudinal tensile force of 3 grams were applied (the highest loading in the study), the pressure contributed less than 13% of the total stress.

**Table 2:** The *α *and *β *coefficients from the longitudinal stress-strain curves as function of luminal pressure in the distal ileum

Coefficient	Luminal pressure (cmH_2_O)				
	
	0	1	2	3	4
*α*	17.3 ± 1.7	21.1 ± 2.9	22.3 ± 2.8	23.6 ± 4.4	38.9 ± 9.3*
*β*	-0.6 ± 0.1	-0.8 ± 0.1*	-1.1 ± 0.1*	-0.9 ± 0.7	-1.9 ± 0.5*

Values are means ± SE. N = 10. *p < 0.05 student *t*-test vs. luminal pressure = 0 cmH_2_O.

## Discussion

The deformation of the intestine during contraction and relaxation is three-dimensional. To obtain a more thorough understanding of the intestinal transport function, we need to know the stresses and strains in multiple directions. The intestinal zero-stress state, the state in which the intestine is stress-free everywhere, serves as the reference state from which the stress-strain relationships can be studied and constitutive equations determined. Our former study provided data on circumferential residual strains and stress-strain relations in the rat small intestine [11]. The current study presents the first data on the zero-stress state and the stress-strain relationship in the intestines in the longitudinal direction. It was found that residual strains occur in the longitudinal direction and vary at different circumferential locations.

### Longitudinal residual strain

The duodenum and ileum were selected for this study due to the differences in structure, function and circumferential mechanical properties as identified previously [11]. The intestine bends outwards in longitudinal direction when cut open in the no-load state, showing a significant longitudinal residual strain. Like the circumferential residual strain [11], the longitudinal mucosal residual strain was negative, indicating the mucosa was compressed longitudinally at the no-load state. The positive residual strain at the serosal side indicated tension in the longitudinal direction. The physiological significance may be the same as for the circumferential residual strain in hollow organs. Prestressing a hollow organ is a mechanism to avoid damage to the inner surface at luminal pressures in a manner similar to the prestressing of mechanical devices. Thus, the compressed mucosa is better protected against injury if there is flow of luminal contents producing high internal pressure than uncompressed mucosa would otherwise be [3]. This protection mechanism may be important in the longitudinal direction because the intestinal wall is exposed to stretching forces during the passage of luminal contents. The longitudinal stress acting in the mucosa decreases due to the effect of prestressing the tissue. The mucosal longitudinal residual strain showed axial variation with larger absolute value observed in the duodenum than in the ileum. It may be related to the thicker mucosal layer and faster passage of food in the duodenum than in the ileum [17]. By comparison, the residual strains in longitudinal direction measured in this study are smaller than those in circumferential direction [11], especially at the mucosal side. This could be due to the largest tensile stress and strain are distributed in the circumferential direction [11] when the intestine is distended during the passage of food. Hence, it is reasonable that larger residual strain exists in circumferential direction.

In order to explore the distribution of longitudinal residual strain around the circumference, seven longitudinal cuts were made to obtain multiple longitudinal strips around the circumference. The longitudinal residual strain in the longitudinal direction was not uniform around the circumference. Larger longitudinal residual strain (both at the mucosal and serosal surface), and larger longitudinal bending angles were found on the mesenteric side and its opposite side in both the duodenum and ileum. Anatomically, they are located where the blood vessels enter the intestinal wall and the capillaries from the ventral and dorsal sides converge. Although significant difference in histology around the circumference has not been identified [18], circumferential variations in structural components such as collagen may exist. The non-homogeneous distribution of the longitudinal residual stress in the circumference of the wall may explain the non-circular shape of the intestine in the resting state.

### Longitudinal stress-strain relations

In general, studies on longitudinal stress-strain properties are few, and in those studies the true zero-stress state was not assessed [12,19]. In this study we found that the neutral axis (the location in the wall where residual strain is zero) was approximately in the middle of the wall. Thus, the deformation (residual strain) in the mid-wall at no-load state was zero. Consequently, the no-load state can be used as the reference when average stresses and strains are computed (assuming a rather thin-walled homogenous wall). It is important to notice that in the intestines the villus of the mucosa does not carry tensile mechanical loads due to the villus structure. The average villus to wall thickness ratio was 0.26/0.61 (unpublished histological data). This was taken into account in the analysis. With the protocol used, the longitudinal wall stress was mainly caused by the longitudinal stretching (87% or more). The curves followed exponential courses. This is consistent with mechanical studies on other biological tissues, in which the passive elastic properties are non-linear and tend to follow exponential courses [13,20]. The *α *constant increased indicating that the intestinal wall became stiffer in the longitudinal direction with luminal pressure.

In conclusion, longitudinal residual strains exist in the small intestine with negative values at the mucosal side and positive values at the serosal side, indicating a longitudinally compressed mucosa and tensile serosa. The longitudinal residual strain showed circumferential variation with the highest values on the mesenteric and anti-mesenteric sides. This indicates that the tissue is not uniform and cannot be treated as a homogenous material. The longitudinal stiffness of the intestinal wall increased with luminal pressure. Longitudinal residual strains must be taken into account in studies of gastrointestinal biomechanical properties.

## Authors' contributions

Yangling Dou participated in the design of the study, carried out the experiments, drafted the manuscript and made the statistical analysis. Yanghua Fan participated in the experiment and carried out the some data analysis. Jingbo Zhao participated in some data analysis and revised the manuscript. Hans Gregersen got the idea for the study, and participated in the design and coordination and helped to draft the manuscript. All authors read and approved the final manuscript.

## References

[B1] Fung YC, Liu SQ (1989). Change of residual strains in arteries due to hypertrophy caused by aortic constriction. Circulation Research.

[B2] Fung YC, Liu SQ (1991). Changes of zero-stress state of rat pulmonary arteries in hypoxic hypertension. Journal of Applied Physiology.

[B3] Gregersen H, Kassab G, Pallencaoe E, Lee C, Chien S, Skalak R, Fung YC (1997). Morphometry and strain distribution in guinea pig duodenum with reference to the zero-stress state. American Journal of Physiology.

[B4] Gregersen H, Kassab G (1996). Biomechanics of the gastrointestinal tract. Neurogastroenterology and Motility.

[B5] Rodriguez EK, Omens JH, Waldman LK, McCulloch AD (1993). Effect of residual stress on transmural sarcomere length distributions in rat left ventricle. American Journal of Physiology.

[B6] Han HC, Fung YC (1996). Direct measurement of transverse residual strains in aorta. American Journal of Physiology.

[B7] Omens JH, Fung YC (1990). Residual strain in rat left ventricle. Circulation Research.

[B8] Costa KD, May-Newman K, Farr D, O'Dell WG, McCulloch AD, Omens JH (1997). Three-dimensional residual strain in midanterior canine left ventricle. American Journal of Physiology.

[B9] Gregersen H, Lee TC, Chien S, Skalak R, Fung YC (1999). Strain distribution in the layered wall of the esophagus. Journal of Biomechanical Engineering.

[B10] Gao C, Gregersen H (2000). Biomechanical and morphological properties in rat large intestine. Journal of Biomechanics.

[B11] Dou Y, Zhao J, Gregersen H (2003). Morphology and stress-strain properties along the small intestine in the rat. Journal of Biomechanical Engineering.

[B12] Zabinski MP, Biancani P (1977). Longitudinal force and stress of rat's esophagus: age-related changes. American Journal of Physiology.

[B13] Fung YC (1993). Biomechanics: Mechanical properties of living tissues.

[B14] Lu X, Gregersen H (2001). Regional distribution of axial strain and circumferential residual strain in the layered rabbit esophagus. Journal of Biomechanics.

[B15] Gregersen H (2002). Biomechanics of the Gastrointestinal Tract.

[B16] Bouchoucha M, Benard T, Dupres M (1999). Temporal and spatial rhythmicity of jejunal wall motion in rats. Neurogastroenterol Mot.

[B17] Christensen J, Johnson LR et al (1987). Motility of the small intestine. Physiology of the gastrointestinal tract.

[B18] Gabella G (1981). On the musculature of the gastrointestinal tract of the guinea pig. Anat Embryol.

[B19] Han HC, Fung YC (1995). Longitudinal strain of canine and porcine aortas. Journal of Biomechanics.

[B20] Dobrin PB (1978). Mechanical properties of arteries. Physiological Reviews.

